# UBE2T-mediated Akt ubiquitination and Akt/β-catenin activation promotes hepatocellular carcinoma development by increasing pyrimidine metabolism

**DOI:** 10.1038/s41419-022-04596-0

**Published:** 2022-02-15

**Authors:** Zhenru Zhu, Chuanhui Cao, Dongyan Zhang, Zhihong Zhang, Li Liu, Dehua Wu, Jingyuan Sun

**Affiliations:** 1 Department of Radiation Oncology, Nanfang Hospital, Southern Medical University, Guangzhou, China; 2 Hepatology Unit and Department of Infectious Diseases, Nanfang Hospital, Southern Medical University, Guangzhou, China

**Keywords:** Liver cancer, Ubiquitylation

## Abstract

The oncogene protein ubiquitin-conjugating enzyme E2T (UBE2T) is reported to be upregulated in hepatocellular carcinoma (HCC) and correlated with poor clinical outcomes of HCC patients. However, the underlying mechanism by which UBE2T exerts its oncogenic function in HCC remains largely unexplored. In this study, in vitro and in vivo experiments suggested that UBE2T promoted HCC development including proliferation and metastasis. GSEA analysis indicated that UBE2T was positively correlated with pyrimidine metabolism, and LC/MS-MS metabolomics profiling revealed that the key products of pyrimidine metabolism were significantly increased in UBE2T-overexpressing cells. UBE2T overexpression led to the upregulation of several key enzymes catalyzing de novo pyrimidine synthesis, including CAD, DHODH, and UMPS. Moreover, the utilization of leflunomide, a clinically approved DHODH inhibitor, blocked the effect of UBE2T in promoting HCC progression. Mechanistically, UBE2T increased Akt K63-mediated ubiquitination and Akt/β-catenin signaling pathway activation. The disruption of UBE2T-mediated ubiquitination on Akt, including E2-enzyme-deficient mutation (C86A) of UBE2T and ubiquitination-site-deficient mutation (K8/14 R) of Akt impaired UBE2T’s effect in upregulating CAD, DHODH, and UMPS. Importantly, we demonstrated that UBE2T was positively correlated with p-Akt, β-catenin, CAD, DHODH, and UMPS in HCC tumor tissues. In summary, our study indicates that UBE2T increases pyrimidine metabolism by promoting Akt K63-linked ubiquitination, thus contributing to HCC development. This work provides a novel insight into HCC development and a potential therapeutic strategy for HCC patients.

## Introduction

Hepatocellular carcinoma (HCC) is the fourth leading cause of cancer-related death worldwide [1]. Due to the rapid growth and distant metastasis, a significant proportion of HCC patients are late staged at diagnosis and miss the optimal chance for surgery [2–4]. Therefore, identifying the key factors involved in the development of HCC and exploring the underlying mechanism is urgently needed.

The oncogene protein ubiquitin-conjugating enzyme E2T (UBE2T) is a ubiquitin-conjugating enzyme (E2) which was widely reported to be upregulated and promotes tumorigenesis, proliferation, and metastasis in various cancers in an E2 activity-dependent manner [5–9]. For example, UBE2T promoted the proliferation of breast cancer cells via ubiquitinating and downregulating BRCA1 [6]. In addition, UBE2T facilitated the degradation of p53 protein via enhancing its ubiquitination, and then increased HCC cell growth [7]. However, the E2 catalytic role of UBE2T and underlying mechanism in modulating HCC proliferation and metastasis is still largely unknown.

Tumor metabolism supplies necessary nutrients to construct neoplasm and maintain cell survival, assisting cancer cells to adapt to the nutrition-lacking tumor microenvironment [10–12]. Increasing evidence has uncovered the importance of pyrimidine metabolism in cancer development by providing necessary precursors used for DNA and RNA biosynthesis. The de novo pyrimidine synthesis pathway is highly coordinated by a series of enzymes including carbamoyl-phosphate synthetase 2, aspartate transcarbamoylase, dihydroorotase (CAD), dihydroorotate dehydrogenase (DHODH), and uridine 5′-monophosphate synthase (UMPS) [13]. Dysregulation of these enzymes by numerous oncogenes and the related signaling pathways was previously shown to elevate pyrimidine synthesis and promote the development in varied types of cancers [14–18]. For example, Yamaguchi et al. found that PCK1 enhanced the metastatic capacity of colorectal cells via activating pyrimidine biosynthesis under hypoxia conditions [16]. In addition, Mathur et al. reported that PTEN-deficiency regulated glutamine flux to pyrimidine synthesis, thus increasing the growth of breast cancer cells [17]. Simultaneously, there have been studies that reported that pyrimidine metabolism correlated with the development and poor prognosis of hepatocellular carcinoma [19–21]. However, the underlying mechanism is rarely explored.

Akt activation and its related signaling pathways play a critical role in regulating varied pathological processes of cancer cells [22–26]. Other than that phosphorylation is well-known to be the active form of Akt, and emerging evidences have established that Lysine (K) 63-linked polyubiquitination is required for Akt membrane localization and full activation, which facilitates the occurrence and development of tumors [27–29]. Although several studies revealed that Akt K63-linked polyubiquitination is modulated by different ubiquitin ligases (E3s) under various stimulations and stresses, its function and the regulatory factors in HCC have not been reported.

In our study, we found that UBE2T plays a key role in HCC development by promoting pyrimidine metabolism, and this process is dependent on UBE2T-mediated Akt K63-linked ubiquitination and Akt/β-catenin signaling pathway activation. Inhibition of pyrimidine metabolism prevents UBE2T-induced HCC progression, highlighting a potential treatment strategy in HCC patients.

## Methods

### Cell culture

HCC-LM3 and MHCC-97H were obtained from the Shanghai Institute of Biochemistry and Cell Biology. Cells were cultured in DMEM (Gibco, USA) supplemented with 10% fetal bovine serum (Gibco, USA). All cells were cultured using standard procedures in a 37 °C humidified incubator with 5% CO_2_.

### Cell proliferation assay

Cells were seeded at a density of 1000/well in 96-well plates. CCK-8 solution (Dojindo Laboratories, Japan) (10 μl/well) was added after at days 0–7. Cells were incubated in the dark for 2 h at 37 °C. The absorbance at 450 nm was measured.

### Migration and invasion assays

Cell migration assay was performed using 8 μm transwell chambers (Falcon, USA). Cell invasion assay was performed using Matrigel-coated chambers (BD Biosciences, USA). After 24 h incubation at 37 °C, the cells on the upper surface of the membrane were removed, the membranes were stained with 0.1% crystal violet for 15 min, and the cells on the lower side were then counted under a microscope.

### Apoptosis flow cytometry, western blot (WB), immunoprecipitation (IP), immunofluorescence (IF) staining, H&E, immunohistochemistry (IHC) staining, RNA extraction, reverse transcription, and quantitative real-time PCR (qRT-PCR)

All these analyses were performed as described previously [30]. Antibody information is shown in Table S1 and the PCR primers were listed in Table S2.

### LC/MS-MS metabolomics profiling

The metabolite extraction from UBE2T-overexpressing and control HCC-LM3 cells were analyzed by Ultra Performance Liquid Chromatography (UPLC, ExionLC AD) and Tandem mass spectrometry (MS/MS, QTRAP^®^). Data analysis was performed using Analyst 1.6.3.

### Mice treatment

BALB/c nude mice (males, 4 to 5 weeks of age) were obtained from the Southern Medical University Animal Resource Center (Guangzhou, China) and grouped randomly. Mice were housed, fed, and monitored in accordance with protocols approved by the Committee for Animal Research at Southern Medical University. Cells (1 × 10^7^) transduced with lentivirus carrying pCMV-luciferase (Genechem Company Ltd., China) were implanted subcutaneously in the mice hind flank to establish tumor growth models or into tail veins to establish tumor metastasis models. Tumor volumes were calculated using a standard formula: length × width^2^/2. The fluorescence intensity of the tumor was detected using an IVIS^@^ Lumina II system (Caliper Life Sciences, USA) before the mice were sacrificed. For Leflunomide (Lef) experiment, mice were intraperitoneally injected with Lef (S1247, Selleck; 7.5 mg/kg) daily until sacrifice.

### RNAi targeting sequence

SiRNAs were synthesized by GenePharma (Suzhou, China). Cells were transfected with the indicated siRNA using Lipofectamine^®^ RNAiMAX (Invitrogen, USA) according to the manufacturer’s instructions. The sequences of siRNAs were as followed:

UBE2T (5′-GCUGACAUAUCCUCAGAAUTT-3′)

AKT (5′-GAACAAUCCGAUUCACGUATT-3′)

β-catenin (5′-GGAUGUUCACAACCGAAUUTT-3′)

NC (5′-CCACACGAGUCUUACCAAGUUGCUU-3′)

### GSEA

The GSEA assay using the TCGA cohort of hepatocellular carcinoma was applied to explore the underlying mechanisms of the effect of UBE2T. The reference gene set was the C2 (c2.cp.kegg.v7.0.symbols.gmt) from the Molecular Signatures Database (MSigDB). UBE2T expression was annotated as a high- or low-UBE2T phenotype. Gene set permutations in each analysis were conducted 1000 times. A gene set was regarded as significantly enriched if a *P* value was less than 0.05.

### Statistics

All data were presented as mean ± standard deviation (SD) from at least triplicate independent experiments. The student’s *t*-test was performed for comparing the differences between the two groups. The comparisons between three or more groups were performed using one-way ANOVA, followed by Tukey’s multiple comparison test. The comparison of mice subcutaneous tumor volume from four groups was analyzed by using a mixed-effects model, followed by Tukey’s multiple comparison test. For all statistical analysis, a *p* value of <0.05 was determined to be significant. All graphs were plotted using GraphPad Prism V.8.

## Results

### UBE2T promotes HCC development in vitro and in vivo

**Fig. 1:**
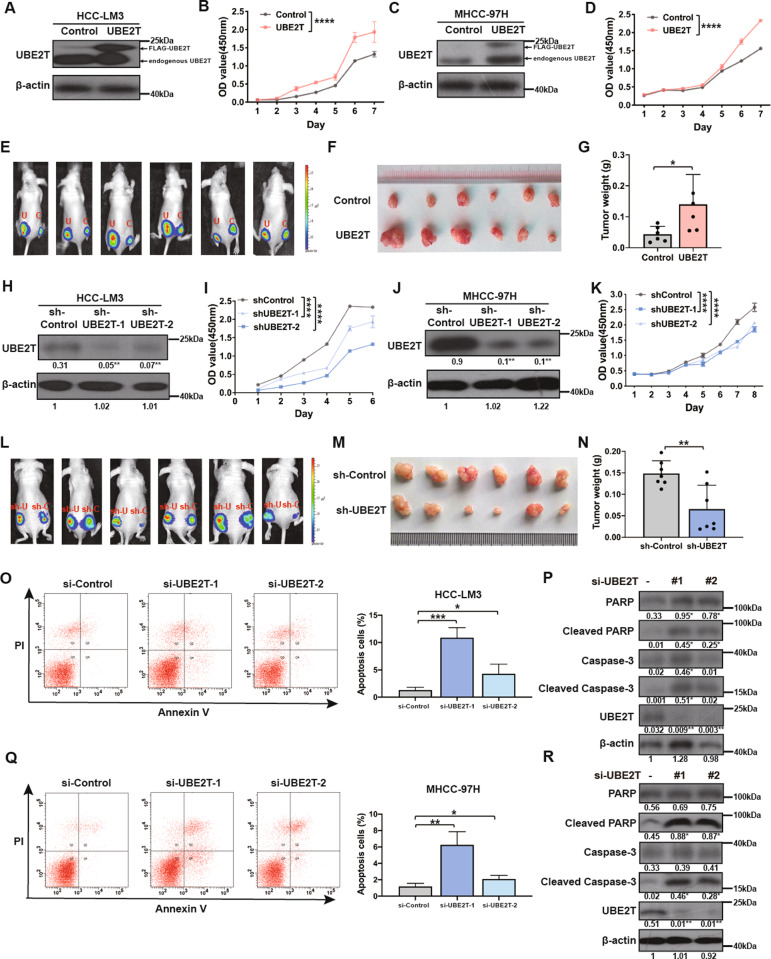
UBE2T promotes HCC proliferation in vitro and in vivo. **A** WB for UBE2T expression in HCC-LM3 cells transduced with UBE2T-overexpressing lentivirus or control lentivirus. **B** The proliferation of UBE2T-overexpressing and control HCC-LM3 cells was assessed by CCK-8 assay. **C** WB for UBE2T expression in MHCC-97H cells transduced with UBE2T-overexpressing lentivirus or control lentivirus. **D** The proliferation of UBE2T-overexpressing and control MHCC-97H cells was assessed by CCK-8 assay. **E** Bioluminescence images of the mice injected subcutaneously with UBE2T-overexpressing and control HCC-LM3 cells. The luminescence signal is represented by an overlaid false-color image with the signal intensity indicated by the scale. **F** The xenografts from panel **E** were shown. **G** Tumor weights of the removed xenografts from panel **E**. **H** WB for UBE2T expression in HCC-LM3 cells transduced with a control shRNA lentiviral vector or two independent shRNA lentiviral vectors targeting UBE2T. **I** The proliferation of UBE2T-silencing and control HCC-LM3 cells was assessed by CCK-8 assay. **J** WB for UBE2T expression in MHCC-97H cells transduced with a control shRNA lentiviral vector or two independent shRNA lentiviral vectors targeting UBE2T. **K** The proliferation of UBE2T-silencing and control MHCC-97H cells was assessed by CCK-8 assay. **L** Bioluminescence images of the mice injected subcutaneously with UBE2T- silencing and control HCC-LM3 cells. **M** The xenografts from panel **L** were shown. **N** Tumor weights of the removed xenografts from panel **L**. **O** The % apoptotic cells of HCC-LM3 cells transfected with siRNA of UBE2T and control was detected by flow cytometry. **P** WB for apoptosis markers in UBE2T-silencing and control HCC-LM3 cells. **Q** The % apoptotic cells of MHCC-97H cells transfected with siRNA of UBE2T and control was detected by flow cytometry. **R** WB for apoptosis markers in UBE2T-silencing and control MHCC-97H cells. In all panels, error bar represent mean ± SD, *P < 0.05, **P < 0.01, ***P < 0.001, ****P < 0.0001. In **B**, **D**, **I**, and **K**, experiments were repeated at least three times, two-way ANOVA was used for comparison between groups. In **G** and **N**, an unpaired two-tailed Student’s *t*-test was used for comparing the two groups. In **O** and **Q**, one-way ANOVA was used for comparison between treatment groups, and Tukey post-hoc test was used for two-group comparisons. The average gray values and the statistical data was shown under the corresponding band. Student’s *t*-test was used for comparisons, *P < 0.05, **P < 0.01.

Previous studies have reported that UBE2T is upregulated in human HCC tissues and a high level of UBE2T is correlated with an unfavorable prognosis of HCC patients [5, 31]. Here, to explore the biological role of UBE2T in HCC development, we firstly performed CCK-8 assays and found that UBE2T overexpression accelerated proliferation in HCC-LM3 and MHCC-97H cells (Fig. 1A–D and Suppl. Figs. 1–5). In addition, we observed that the subcutaneous xenografts derived from HCC-LM3 and MHCC-97H cells with UBE2T overexpression displayed quicker tumor growth and heavier tumor weight compared with those derived from control cells (Fig. 1E–G and Suppl. Fig. 6). In contrast, knockdown of UBE2T attenuated HCC proliferation both in vitro and in vivo (Fig. 1H–N and Suppl. Figs. 1–5). Since apoptosis is closely related to cell survival, by conducting flow cytometry for the percentage of apoptotic cells and WB for apoptosis-related proteins, we found that UBE2T silencing promoted apoptosis in HCC cells (Fig. 1O–R and Suppl. Figs. 1–5).

**Fig. 2:**
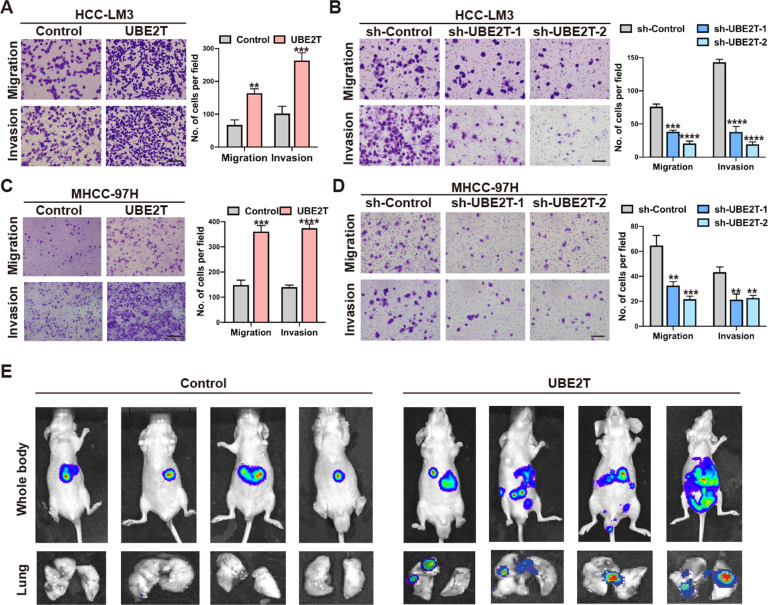
UBE2T promotes HCC metastasis in vitro and in vivo. **A**–**D** Transwell assays to assess the migration and invasion ability of **A** UBE2T-overexpressing and control HCC-LM3 cells, **B** UBE2T-silencing and control HCC-LM3 cells, **C** UBE2T-overexpressing and control MHCC-97H cells, **D** UBE2T-silencing and control MHCC-97H cells. Quantifications of cell number per fields are from at least three independent experiments. Error bar represent mean ± SD, *P < 0.05, **P < 0.01, ***P < 0.001, ****P < 0.0001. Scale bar = 100 μm. In **A** and **C**, a two-tailed Student’s *t*-test was used for comparing the two groups. In **B** and **D**, one-way ANOVA was used for comparison between treatment groups, and Tukey post hoc test was used for two-group comparisons. **E** UBE2T-overexpressing and control HCC-LM3 cells were injected in nude mice by tail vein. Bioluminescence images were collected at 30 days. The luminescence signal is represented by an overlaid false-color image with the signal intensity indicated by the scale.

Next, we explored the metastatic ability of HCC cells with different UBE2T expression levels. We found that migration and invasion of HCC cells were increased by UBE2T overexpression, while reduced by UBE2T knockdown (Fig. 2A–D and Suppl. Fig. 7a, b). Given that epithelial-mesenchymal transition (EMT) is involved in cancer metastasis, we examined the protein levels of EMT markers and found that Vimentin was upregulated, while E-cadherin was downregulated by UBE2T overexpression (Suppl. Figs. 1–5 and Suppl. Fig. 7c). By contrast, UBE2T-knockdown cells displayed a higher expression level of E-cadherin and a lower level of Vimentin than control cells (Suppl. Figs. 1–5, 7d). Consistently, by injecting UBE2T-overexpressing or control HCC-LM3 cells intravenously into nude mice, we observed more liver and lung metastasis in the UBE2T-overexpressing group (Fig. 2E and Supple. Fig. 8).

### UBE2T increases pyrimidine metabolism of HCC cells

**Fig. 3:**
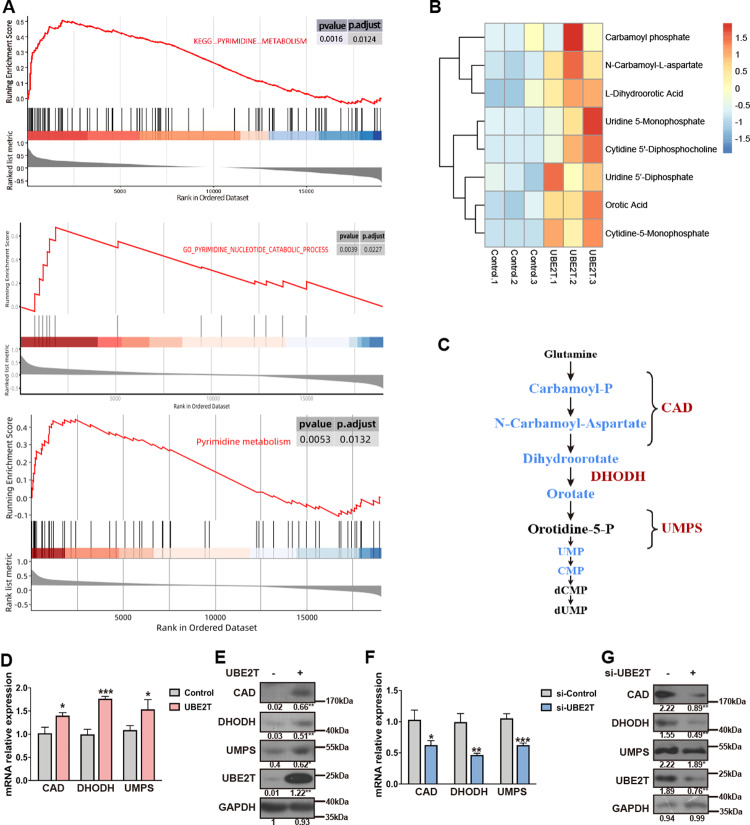
UBE2T regulates pyrimidine metabolism in HCC cells. **A** Results of GSEA were plotted to visualize the correlation between the expression of UBE2T and gene signatures of pyrimidine metabolism in the TCGA cohort. **B** LC-MS/MS-based metabolomics profiling analysis was conducted to analyze UBE2T-overexpressing and control HCC-LM3 cells, and the results of unbiased hierarchical clustering was presented. **C** Schematic of the de novo pyrimidine synthesis pathway. **D** The mRNA levels of the indicated genes were detected by qRT-PCR analysis in UBE2T-overexpressing and control HCC-LM3 cells. **E** The protein levels of the indicated genes were detected by WB analysis in UBE2T-overexpressing and control HCC-LM3 cells. **F** The mRNA levels of the indicated genes were detected by qRT-PCR analysis in UBE2T-silencing and control MHCC-97H cells. **G** The protein levels of the indicated genes were detected by immunoblotting analysis in UBE2T-silencing and control MHCC-97H cells. All values shown are mean ± SD of triplicate measurements and experiments, *P < 0.05, **P < 0.01, ***P < 0.001, ****P < 0.0001. In **D** and **F**, a two-tailed Student’s *t*-test was used for comparing the two groups. The average gray values and the statistical data were shown under the corresponding band. Student’s *t*-test was used for comparisons, *P < 0.05, **P < 0.01.

Next, to explore how UBE2T modulates HCC development, we performed gene set enrichment analysis (GSEA) and found that UBE2T was positively correlated with pyrimidine metabolism (Fig. 3A). In addition, KEGG pathway analysis further indicated the potential regulatory role of UBE2T in pyrimidine metabolism (Suppl. Fig. 9a). To confirm this observation, we used LC-MS/MS-based metabolomics profiling analysis to examine the effect of UBE2T on pyrimidine metabolism. By performing unsupervised hierarchical clustering analysis on the differentially expressed pyrimidine metabolite profiles between UBE2T-overexpressing and control cells, we found that the most salient finding was a significant increase of main products of pyrimidine metabolism in UBE2T-overexpressing cells (Fig. 3B, C and Suppl. Fig. 9b–i). Correspondingly, UBE2T overexpression elevated the levels of several key enzymes involved in de novo pyrimidine synthesis, including CAD, DHODH, and UMPS on both mRNA and protein levels (Fig. 3D, E and Suppl. Figs. 1–5). While knockdown of UBE2T reduced the levels of these enzymes (Fig. 3F, G and Suppl. Figs. 1–5). Together, these results indicated that UBE2T increases pyrimidine metabolism.

### Leflunomide impairs UBE2T-mediated HCC development

**Fig. 4:**
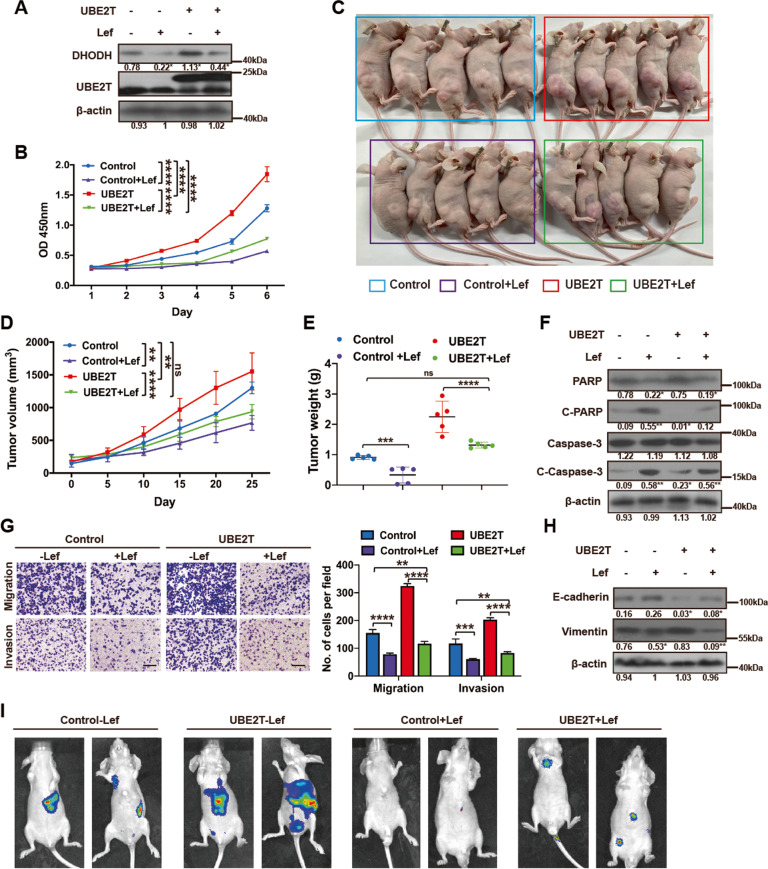
Leflunomide impairs UBE2T-induced proliferation and metastasis in HCC. **A** Total cells lysate from UBE2T-overexpressing and control HCC-LM3 cells treated with or without Lef (50 μM, 48 h) were analyzed by WB. **B** Cells were treated as panel (**A**). Cell survival was assessed by CCK-8 assay. Experiments were repeated at least three times. **C** Transplanted xenografts were established with UBE2T-overexpressing (UBE2T) and vector transduced (Control) HCC-LM3 cells. The tumor-bearing mice were treated with or without Lef (7.5 mg/kg/d) by intraperitoneal injection. **D** Tumor volumes from each group were tracked for 25 days. *N* = 5 in each group. **E** Tumor weights of the removed xenografts from each group were measured. **F** Total cells lysate from the cells treated as panel (**A**) were analyzed for the apoptosis markers by WB. **G** Transwell assay to assess the migration and invasion ability of the cells treated as panel (**A**). Quantification of cell numbers per fields is from at least three independent experiments. Scale bar = 100 μm. **H** Total cells lysate from the cells treated as panel (**A**) were analyzed for the EMT markers by WB. **I** UBE2T-overexpressing and control HCC-LM3 cells were injected in nude mice by tail vein. The tumor-bearing mice were treated with or without Lef (7.5 mg/kg/d) by intraperitoneal injection. Bioluminescence images were collected at 30 days. The luminescence signal is represented by an overlaid false-color image with the signal intensity indicated by the scale. All values shown are mean ± SD. *P < 0.05, **P < 0.01, ***P < 0.001, ****P < 0.0001. In **B**, **E**, and **G**, one-way ANOVA was used for comparison between treatment groups, and Tukey post hoc test was used for two-group comparisons. In **D**, two-way ANOVA was used for comparison between groups. The average gray values and the statistical data were shown under the corresponding band. Student’s *t-*test was used for comparisons, *P < 0.05, **P < 0.01.

In the pathway of de novo pyrimidine synthesis, DHODH is a rate-limiting enzyme, which catalyzes the conversion of dihydroorotate to orotate. Leflunomide (Lef), a DHODH inhibitor, is a commercially available agent approved by US Food and Drug Administration (FDA) for rheumatoid arthritis treatment. Therefore, to determine whether UBE2T-mediated HCC development relies on pyrimidine metabolism, we treated the UBE2T-overexpressing and control HCC cells with Lef. In line with previous reports, Lef reduced DHODH level and attenuated the UBE2T-induced upregulation of DHODH (Fig. 4A and Suppl. Figs. 1–5). Cell viability assays showed that Lef reduced the proliferation of UBE2T-overexpressing cells (Fig. 4B). Consistently, compared with vehicle treatment, challenging tumor-bearing mice with Lef caused slower tumor growth and lighter tumor weight of the xenografts derived from UBE2T-overexpressing HCC cells (Fig. 4C–E). Accordingly, Lef caused lower levels of apoptosis markers in UBE2T-overexpressing cells (Fig. 4F and Suppl. Figs. 1–5). Furthermore, Lef reduced the effect of UBE2T on promoting metastasis of HCC cells shown by transwell assays and EMT marker detection (Fig. 4G, H and Suppl. Figs. 1–5). Additionally, Lef substantially impaired the lung and liver colonization of UBE2T-overexpressing HCC cells in vivo (Fig. 4I).

### UBE2T activates Akt/β-catenin signaling pathway

**Fig. 5:**
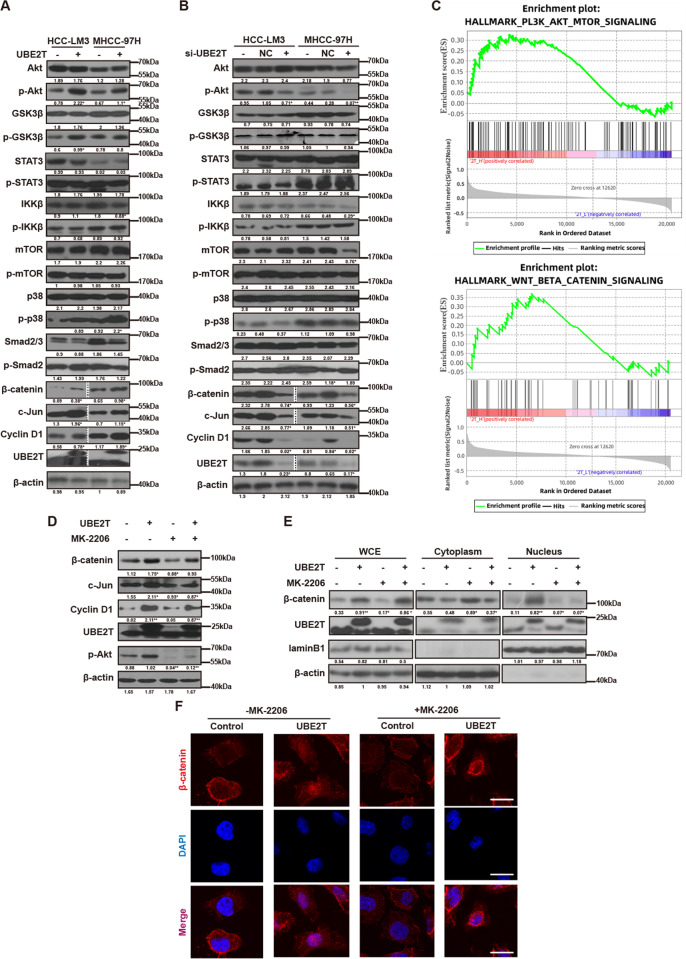
UBE2T activates the Akt/β-catenin signaling pathway. **A**, **B** WB was used to assess the key factors in several signaling pathways in **A** UBE2T-overexpressing and control, **B** UBE2T-silencing and control HCC-LM3/MHCC-97H cells. **C** GSEA exhibited the correlation between the expression of UBE2T and gene signatures of the Akt/β-catenin signaling pathway in the TCGA cohort. **D** UBE2T-overexpressing and control HCC-LM3 were treated with or without MK-2206 (50 μM, 48 h). Total cells lysate were detected for the indicated proteins. **E** cells were treated as (**D**) and then collected for subcellular protein extraction, followed by WB to detect β-catenin and p-Akt. **F** Cells were treated as panel (**D**). Representative images of immunofluorescence staining for β-catenin (Red) are shown. Scale bar = 200 μm. The average gray values and the statistical data were shown under the corresponding band. Student’s *t*-test was used for comparisons, *P < 0.05, **P < 0.01.

To explore which signaling pathway participates in UBE2T-mediated pyrimidine metabolism, we screened the key factors involved in several widely studied signaling pathways in cells with different UBE2T levels by western blot, respectively. Results showed that UBE2T overexpression led to the increase in p-Akt, β-catenin, and downstream targets of β-catenin, including c-Jun and Cyclin D1 (Fig. 5A and Suppl. Figs. 1–5). Whereas UBE2T knockdown decreased the levels of these proteins (Fig. 5B and Suppl. Figs. 1–5). Besides, GSEA analysis also indicated that UBE2T is positively correlated with Akt and β-catenin signaling pathways (Fig. 5C). β-catenin is one of the downstream effectors of Akt, and to test whether UBE2T activates β-catenin via Akt, we then treated UBE2T-overexpressing HCC-LM3 cells with Akt inhibitor MK-2206 and siRNA specifically targeting Akt. We found that MK-2206 and si-Akt impaired the role of UBE2T in upregulating the levels of β-catenin, c-Jun, and Cyclin D1 (Fig. 5D and Suppl. Figs. 1–5, 10a). Since the translocation of β-catenin from the cytosol to nucleus is the indicator for β-catenin activation, we then examined the effect of MK-2206 and si-Akt on β-catenin subcellular localization by WB and IF staining. Results showed that MK-2206, as well as si-Akt, weakened the effect of UBE2T in promoting β-catenin nuclear accumulation (Fig. 5E, F and Suppl. Figs 1–5, 10b, c). These results suggested that UBE2T activates β-catenin via Akt.

### UBE2T is required for Akt K63-linked ubiquitination and activation

**Fig. 6:**
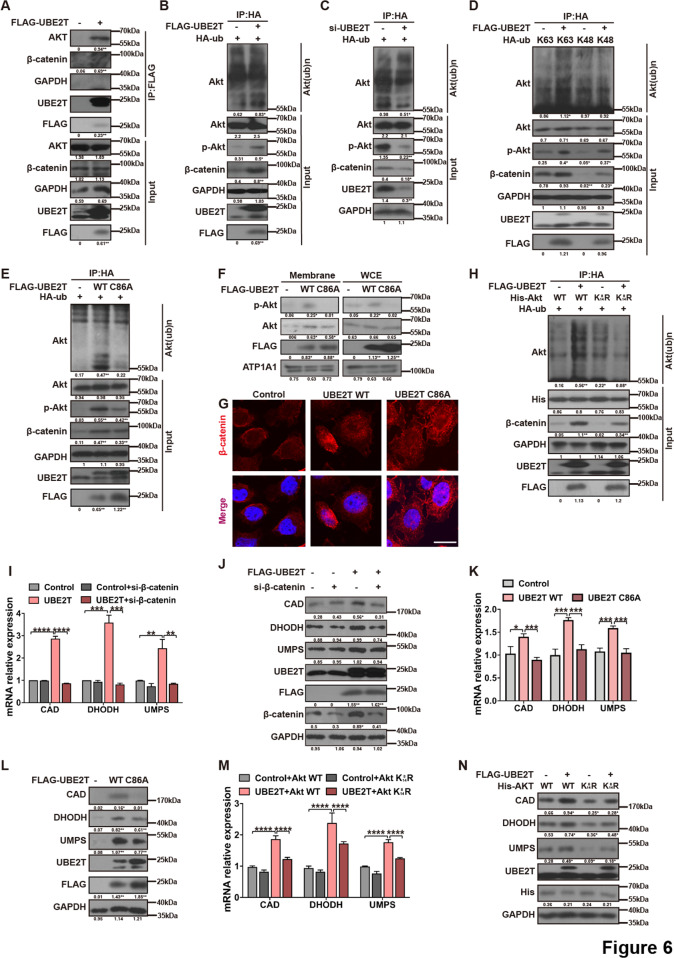
UBE2T activates the Akt/β-catenin pathway via regulating Akt K63-ubiquitination and leads to upregulation of key enzymes involved in de novo pyrimidine metabolism. **A** HCC-LM3 cells were transfected with the FLAG or FLAG-UBE2T vector. FLAG-UBE2T complexes were purified by using an anti-FLAG antibody and analyzed by the indicated antibodies. **B** UBE2T-overexpressing and control HCC-LM3 cells were transfected with HA-ub vector and treated with MG132 (5 μM) for 8 h. IP using anti-HA antibody followed by WB to detect Akt ubiquitination. **C** UBE2T-silencing and control MHCC-97H cells were transfected with HA-ub vector and treated with MG132 (5 μM) for 8 h. IP using anti-HA antibody followed by WB to detect Akt ubiquitination. **D** 293 T cells were co-transfected with FLAG-UBE2T and HA-K48-ub or HA-K63-ub, then treated with MG132 (5 μM) for 8 h. Ubiquitinated AKT was detected in HA immunoprecipitation. **E** 293 T cells were co-transfected with HA-ub and FLAG-UBE2T WT or FLAG-UBE2T C86A, then treated with MG132 (5 μM) for 8 h. The whole-cell extracts were collected for IP using an anti-HA antibody followed by WB for Akt ubiquitination. **F** Whole-cell extract and membrane fraction from HCC-LM3 cells transfected with FLAG-UBE2T WT or FLAG-UBE2T C86A were analyzed for Akt by WB. ATP1A1 was included as a loading control for membrane protein. **G** Cells were treated as panel (**F**). Representative images of IF staining for β-catenin (Red) are shown. Scale bar = 200 μm. **H** 293 T cells were transfected with various constructs as indicated, then treated with MG132 (5 μM) for 8 h and performed for Akt ubiquitination analysis. **I** The mRNA levels of the indicated genes were detected by qRT-PCR analysis in UBE2T-silencing and control HCC-LM3 cells transfected with siRNA targeting β-catenin. **J** HCC-LM3 cells were co-transfected with FLAG-UBE2T and siRNA targeting β-catenin and then collected for WB**. K**, **L** HCC-LM3 cells were transfected with FLAG-UBE2T WT or FLAG-UBE2T C86A and collected for qRT-PCR (**K**) and WB (**L**). **M** The mRNA levels of the indicated genes were detected by qRT-PCR analysis in UBE2T-silencing and control HCC-LM3 cells transfected with His-Akt WT or His-Akt K8/14 R. **N** HCC-LM3 cells were transfected with FLAG-UBE2T, and His-Akt WT or His-Akt K8/14 R, then collected for WB. In **I**, **K**, and **M**, all data in graph bars are mean ± SD from triplicate experiments. *P < 0.05, **P < 0.01, ***P < 0.001, ****P < 0.0001 by one-way ANOVA followed by Tukey post hoc test. The average gray values and the statistical data were shown under the corresponding band. Student’s *t-*test was used for comparisons, *P < 0.05, **P < 0.01.

To clarify the activating role of UBE2T on the Akt/β-catenin signaling pathway is direct or indirect, we performed IP to test whether UBE2T interacts with Akt or β-catenin. Results showed that UBE2T was associated with Akt but not β-catenin (Fig. 6A and Suppl. Figs. 1–5). Since UBE2T is an E2 enzyme, we hypothesized that UBE2T might bind with and ubiquitinate Akt. To test this, we assessed the ubiquitination of Akt in UBE2T-overexpressing cells and found that UBE2T promoted Akt polyubiquitination, in company with increased Akt phosphorylation and β-catenin (Fig. 6B and Suppl. Figs. 1–5, 11a). Consistently, Akt polyubiquitination, activation, and β-catenin level were reduced in UBE2T-knockdown cells (Fig. 6C and Suppl. Figs. 1–5, 11b). It has been well demonstrated that Akt ubiquitination through the K48-ubiquitin chain promotes Akt for proteasomal degradation, while the K63-ubiquitin chain leads to Akt membrane recruitment, phosphorylation, and activation. To identify the way how UBE2T ubiquitinates Akt, we co-expressed a FLAG-Akt with either an HA-tagged K48 or K63 only ubiquitin mutant (ubiquitin can only be added to the K48 or K63 site) in 293 T cells and found that Akt was modified by K63-linked ubiquitination (Fig. 6D and Suppl. Figs. 1–5, 11c). Meanwhile, K63 but not K48-mediated Akt ubiquitination increased the level of phosphorylated Akt and β-catenin (Fig. 6D). Moreover, though blocking the ubiquitin-proteasome system with MG132 increased the expression of β-catenin and p-Akt in HCC-LM3 cells, UBE2T overexpression further activated the Akt/β-catenin axis (Suppl. Figs 1–5, 11d).

Next, we detected whether UBE2T-mediated Akt K63-ubiquitination is required for Akt activation. Firstly, we found that the UBE2T C86A mutant, which is lack of E2 activity, abolished Akt K63-ubiquitination, and Akt activation shown by less Akt/p-Akt membrane recruitment, and β-catenin nuclear accumulation (Fig. 6E–G and Suppl. Figs. 1–5, 11e). In addition, reduced Akt K63-linked ubiquitination and Akt/β-catenin signaling activation were also observed in the cells carrying the mutation of K8 and K14, which are two key residues for Akt K63-linked ubiquitination (Fig. 6H and Suppl. Figs. 1–5, 11f). Together, these data suggested that UBE2T activates the Akt/β-catenin signaling pathway by mediating the K63-linked ubiquitination of Akt.

### UBE2T upregulates de novo pyrimidine synthesis-related enzymes via activating Akt/β-catenin

Our previous data showed that UBE2T upregulated the levels of CAD, DHODH, and UMPS. Next, we determined the regulatory role of UBE2T-mediated Akt/β-catenin activation on these de novo pyrimidine synthesis-related enzymes. Firstly, we found that knockdown of β-catenin remarkably decreased UBE2T-induced upregulation of CAD, DHODH, and UMPS on both mRNA and protein levels (Fig. 6I, J and Suppl. Figs. 1–5), suggesting that the effect of UBE2T on upregulating these enzymes relies on β-catenin. Moreover, the disruption of UBE2T-mediated ubiquitination on Akt, including UBE2T C86A and Akt K8/14 R attenuated UBE2T’s effect in upregulating CAD, DHODH, and UMPS (Fig. 6K–N and Suppl. Figs. 1–5). Together, these results indicated that UBE2T upregulates de novo pyrimidine synthesis-related enzymes via ubiquitinating Akt and activating Akt/β-catenin.

### The axis of UBE2T/Akt/β-catenin is correlated with pyrimidine metabolism in HCC

**Fig. 7:**
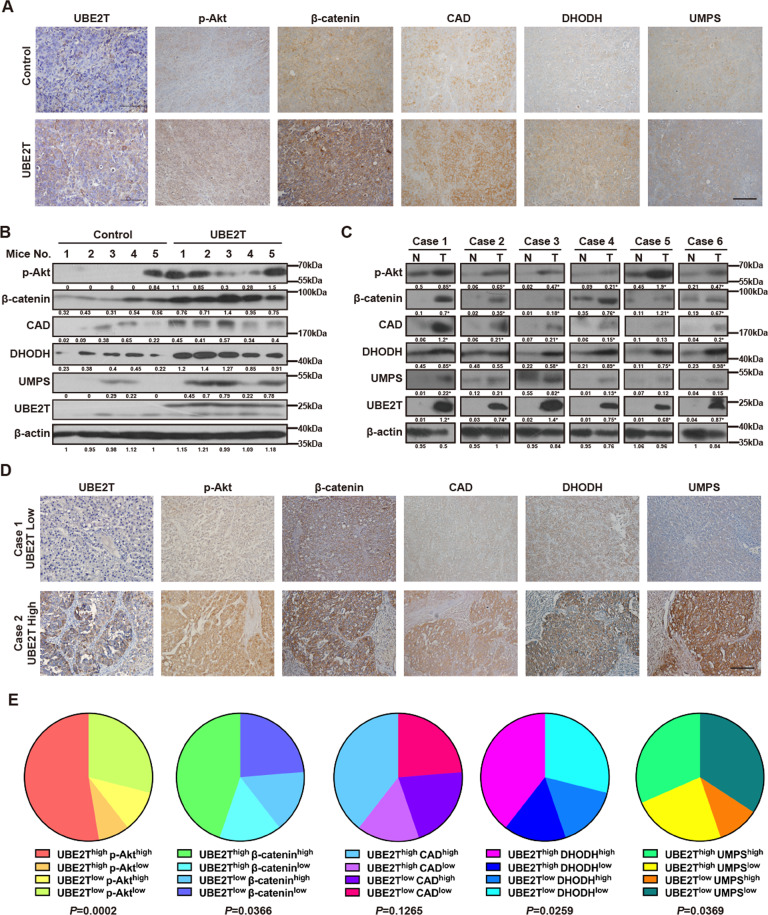
UBE2T positively correlates with the Akt/β-catenin signaling pathway and de novo pyrimidine metabolism-related enzymes in HCC. **A** The representative images of IHC staining for the indicated proteins in xenografts derived from UBE2T-overexpressing or control HCC-LM3 cells. Scale bar = 100 μm. **B** WB was performed to detect the indicated proteins in xenografts derived from UBE2T-overexpressing or control HCC-LM3 cells. **C** WB of the indicated protein in six pairs of HCC tissues (T) and matched non-tumorous liver tissues (N) was performed. **D** Representative cases of HCC specimens with high level or low levels of UBE2T from 38 HCC patients were analyzed by IHC staining with the indicated proteins. Scale bar = 100 μm. **E** The expression of the indicated proteins in 38 HCC specimens were analyzed by IHC analysis. The relative proportions of protein expressions were illustrated as a pie chart. *P < 0.05, **P < 0.01, ***P < 0.001, ****P < 0.0001 by chi-square test. The average gray values and the statistical data were shown under the corresponding band. Student’s *t*-test was used for comparisons, *P < 0.05, **P < 0.01.

To determine whether the expression levels of UBE2T, p-Akt, β-catenin, CAD, DHODH, and UMPS were correlated in HCC, we harvested the xenografts derived from UBE2T-overexpressing or control HCC-LM3 cells. The IHC staining and WB results showed that UBE2T overexpression increased the expression of p-Akt, β-catenin, CAD, DHODH, and UMPS (Fig. 7A, B and Suppl. Figs. 1–5).

In addition, we also collected 38 pairs of HCC and adjacent non-tumorous tissues and found that the patients with higher UBE2T tended to have higher levels of p-Akt, β-catenin, CAD, DHODH, and UMPS (Fig. 7C, D and Suppl. Figs. 1–5). Moreover, systematical analysis of IHC images revealed the statistically positive correlations between UBE2T and p-Akt, β-catenin, DHODH, UMPS (Fig. 7E). Although there was a trend that HCC patients with high levels of UBE2T exhibited upregulated levels of CAD, the difference did not reach significance. Nevertheless, our findings implied the positive relation between UBE2T, Akt/β-catenin, and pyrimidine metabolism.

## Discussion

**Fig. 8:**
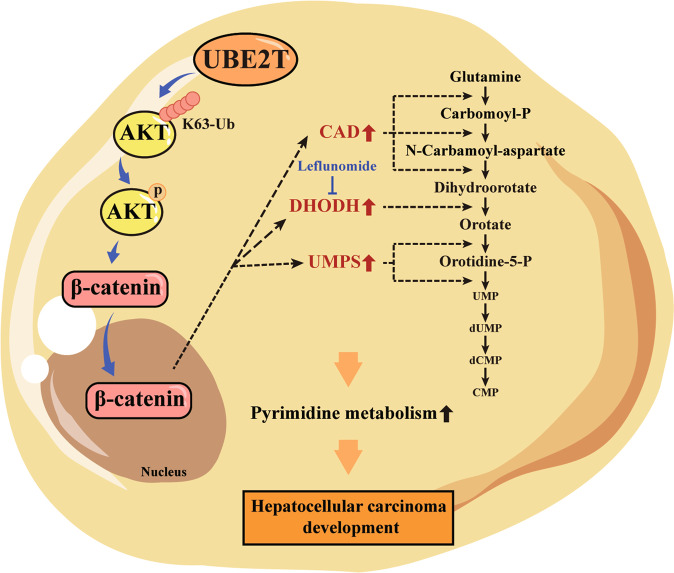
The schematic of UBE2T-mediated regulation of pyrimidine metabolism and HCC development. UBE2T upregulates Akt K63-ubiquitination and then promotes Akt phosphorylation and activation, thus leading to the nuclear translocation of β-catenin. The UBE2T-induced Akt/β-catenin axis activation increases the expression of de novo pyrimidine synthesis-related enzymes, including CAD, DHODH, and UMPS, which facilitates the pyrimidine metabolism and hepatocellular carcinoma development. The oncogenic role of UBE2T was impaired by Leflunomide, which is an inhibitor of DHODH.

In this study, we determined the novel role of UBE2T in promoting HCC development by facilitating pyrimidine metabolism, exhibited by upregulated de novo pyrimidine-related enzymes and increased pyrimidine metabolism products. This occurs by UBE2T regulating the K63-ubiquitination and phosphorylation of Akt, leading to the nuclear translocation of β-catenin, which upregulates the expression of de novo pyrimidine synthesis-related enzymes (Fig. 8). Cancer cells require high levels of pyrimidine products to sustain cell DNA biosynthesis and drive cell cycle progression, which indicates that targeting de novo pyrimidine synthesis could be a potential strategy for cancer therapy [14, 32, 33]. Lef, as the inhibitor of DHODH, has been reported by several pre-clinical studies to exert an antitumor effect on several types of cancers [16, 34–40]. For example, Mathur et al. reported that Lef interrupted de novo pyrimidine synthesis-dependent glutamine generation and caused lethality in PTEN-mutation cells [17]. In addition, Yamaguchi et al. revealed that PCK1 enhanced colorectal cancer metastasis by driving pyrimidine nucleotide biosynthesis under hypoxia and Lef impaired the pro-metastasis role of PCK1 [16]. These findings suggested that the active pyrimidine metabolism caused by several oncogenes might generate vulnerability to Lef treatment and the utilization of Lef might be a potential anti-cancer agent, especially for those patients with higher levels of pyrimidine metabolism. UBE2T has been shown to be upregulated in HCC based on the TCGA database and several clinical cohorts. Moreover, the amplification rate of UBE2T in HCC patients reaches up to about 12% [41]. Here, we showed that UBE2T promoted pyrimidine metabolism, and the level of UBE2T was positively related with the levels of key enzymes involved in de novo pyrimidine synthesis. Thus, Lef might benefit a certain portion of HCC patients in the clinical scenario.

UBE2T plays a critical role in varied cancers by ubiquitinating and degrading some cancer suppressors. For example, Ueki et al. reported that UBE2T led to polyubiquitination and degradation of BRCA1, which is a well-known tumor suppressor in breast cancer [6]. Additionally, p53 is a classic tumor suppressor in HCC, and p53 mutation is considered to be one of the driver genes in HCC and occurs in about 20–30% of HCC patients [42–44]. UBE2T was demonstrated to suppress the expression of p53 by increasing the ubiquitination of p53 [7]. Although in these studies, the way how ubiquitin was linked to substrates by UBE2T was not mentioned, we assumed that it may be K48-linked ubiquitination according to the substrate consequence. Unlike the K48-linked ubiquitin chain generally targets proteins for degradation via 26S proteasome, ubiquitination through K63 is established to play a critical role in signaling pathway transduction, protein trafficking, and DNA damage repair [45–49]. Although numerous reports have shown that UBE2T activated Akt signaling pathway, there is still a gap existing between UBE2T and Akt activation [50–52]. In our study, for the first time, we uncovered a direct evidence that UBE2T activates the Akt/β-catenin signaling pathway by interacting with Akt and facilitating Akt K63-linked ubiquitination. In consistent, Yu et al. reported that in gastric cancer, UBE2T ubiquitinated RACK1, a member of the degradation complex of β-catenin, resulted in the activation and translocation of β-catenin, which further supports our conclusions about the connection between UBE2T and β-catenin [53].

As one of the key factors for cell survival and metabolism reprogramming involved in cancer development, Akt undergoes many kinds of post-translational modifications by various enzymes [54]. Recent studies have uncovered the essential roles of K63-linked ubiquitination by different enzymes on K8 and K14 within the PH domain for Akt activation under varied conditions. For example, in response to EGF stimulation, Skp2, which is a subunit of the Skp1-Cullin-1-F-box (SCF) ubiquitin E3 ligase complex, is required for Akt K63-linked ubiquitination, membrane localization, and activation [55]. While, upon the trigger by IGF, another E3 ligase, TRAF6 is engaged in Akt K63-linked ubiquitination and activation [27]. However, the E2 enzymes involved in Akt K63-linked ubiquitination is largely unknown. By now, UBC13 is the only known E2 that works with TRAF6 and promotes Akt K63-linked polyubiquitination [28, 54, 56]. In our study, we revealed a novel E2, UBE2T increased Akt K63-linked polyubiquitination, membrane accumulation, and activation. However, the E3 ligase which UBE2T cooperates with to ubiquitinate Akt was not demonstrated in our current study and is worthy to be explored in the future.

Although it is widely accepted that E3 ligases recognize specific substrates, E2s determine the type of the ubiquitin chain synthesizing on the substrates. However, some new emerging evidence have shown that one E2 might mediate varied types of ubiquitin chain linkage on substrates and execute different functional roles. For example, UBC13, which is a major E2 known to trigger K63-linked ubiquitination with the assistance of its cofactor UEV1A, mediated ubiquitination and degradation of Sirt1 in CRC cells [57–60]. Although how the ubiquitin chain was linked to Sirt1 by UBC13 has not been discussed, based on the consequence, we assumed that UBC13 promoted K48 but not K63-ubiquitination of Sirt1. Moreover, besides ubiquitinating and degrading substrates, such as p53 and BRCA1, UBE2T also monoubiquitinates and catalyzes FANCD2 for DNA inter-strand crosslink damage repair [61–63]. We previously reported that UBE2T induces HCC radioresistance by monoubiquitinating H2AX [41]. Here, in this study, we revealed another novel function of UBE2T on ubiquitinating Akt via K63 linkage.

Accumulating evidence have shown that Akt K63-linked ubiquitination is critically involved in cell metabolism. For example, Chan et al. reported that Skp2 regulates glycolysis of breast cancer cells through promoting Akt K63-ubiquitination and activation [27]. In addition, Yu et al. showed that Skp2 promotes HK2 mitochondrial localization via upregulation of Akt K63-ubiquitination and activation, thus increasing HK2-mediated glycolysis in nasopharyngeal carcinoma [55]. Besides glycolysis, current knowledge about the effect of Akt K63-ubiquitination on other cell metabolism of cancer cells is still limited. Here, we uncovered that UBE2T-mediated Akt K63-ubiquitination is required for de novo pyrimidine synthesis.

Numerous upstream regulators involved in pyrimidine metabolism have been proposed previously. For example, PTEN-deficiency was proved to drive glutamine flux through the de novo pyrimidine synthesis pathway [17]. It is well established that PTEN is a negative regulator of the phosphatidylinositol-3-kinase (PI3K)/Akt signaling pathway [64, 65]. Analogously, in this study, we found that Akt activation increased de novo pyrimidine synthesis in HCC cells. These studies implied that PTEN/Akt may be a potent signaling cascade in activating de novo pyrimidine synthesis. Furthermore, the activation of mTORC1 led to phosphorylates S1859 on CAD, the enzyme that catalyzes the first three steps of de novo pyrimidine, by ribosomal protein S6 kinase 1 (S6K1) in mouse embryo fibroblasts (MEFs) [66]. mTOR and β-catenin are reported to be the two major effectors of Akt, and we found that UBE2T-mediated pyrimidine metabolism stimulation via Akt/β-catenin but not Akt/mTOR signaling pathway activation in HCC. These reports indicated that Akt is a central factor in modulating pyrimidine metabolism, and the downstream effectors of Akt involved might vary under different conditions.

In summary, we demonstrated that UBE2T increases Akt K63-ubiquitination and promotes Akt activation, resulting in the upregulation of the de novo pyrimidine synthesis-related enzymes and the stimulation of pyrimidine metabolism. Utilization of pyrimidine metabolism inhibitor Lef significantly impaires UBE2T-mediated HCC development and progression. Our work highlights a potential treatment strategy for targeting UBE2T/Akt/β-catenin signaling pathway-mediated pyrimidine metabolism in HCC.

## Supplementary information

supplementary materials

author contribution

checklist

## Data Availability

All data relevant to the study are included in the article or uploaded as supplementary information. The data generated and/or analyzed during the current study are available on reasonable request from the corresponding author.
