# Supplementary material for: UBE2T-mediated Akt ubiquitination and Akt/β-catenin activation promotes hepatocellular carcinoma development by increasing pyrimidine metabolism
Source: Cell Death Dis. 2022 Feb 15;13(2):154. doi: 10.1038/s41419-022-04596-0 (PMC8847552; doi:10.1038/s41419-022-04596-0)
Supplement: Supplementary file 1 — supplementary materials [file 41419_2022_4596_MOESM1_ESM.docx]

**Supplementary Figures and Tables**

**Supplementary Figures 1-5 are unedited gels for all the western blot bands in the article**


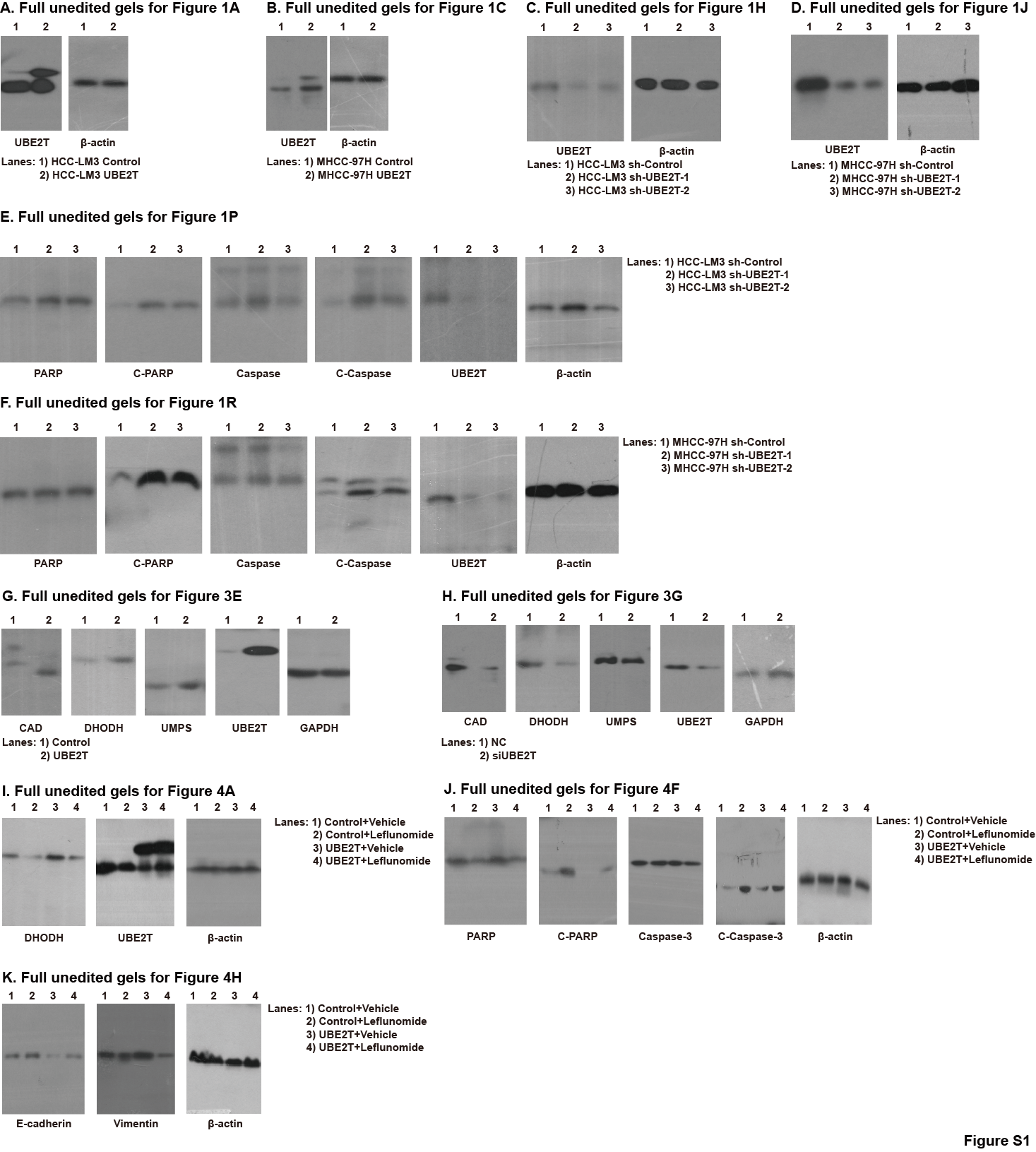


**
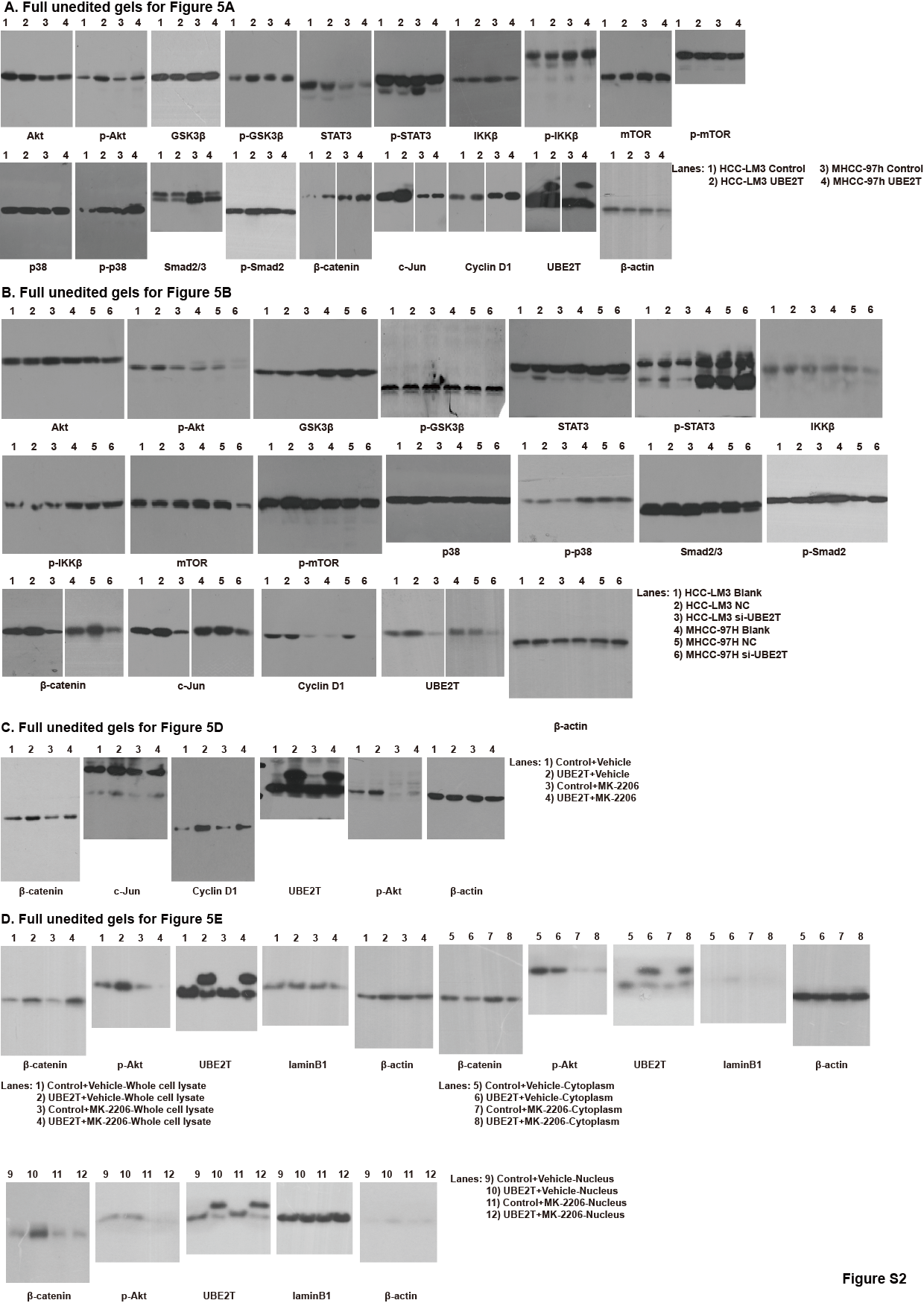
**

**
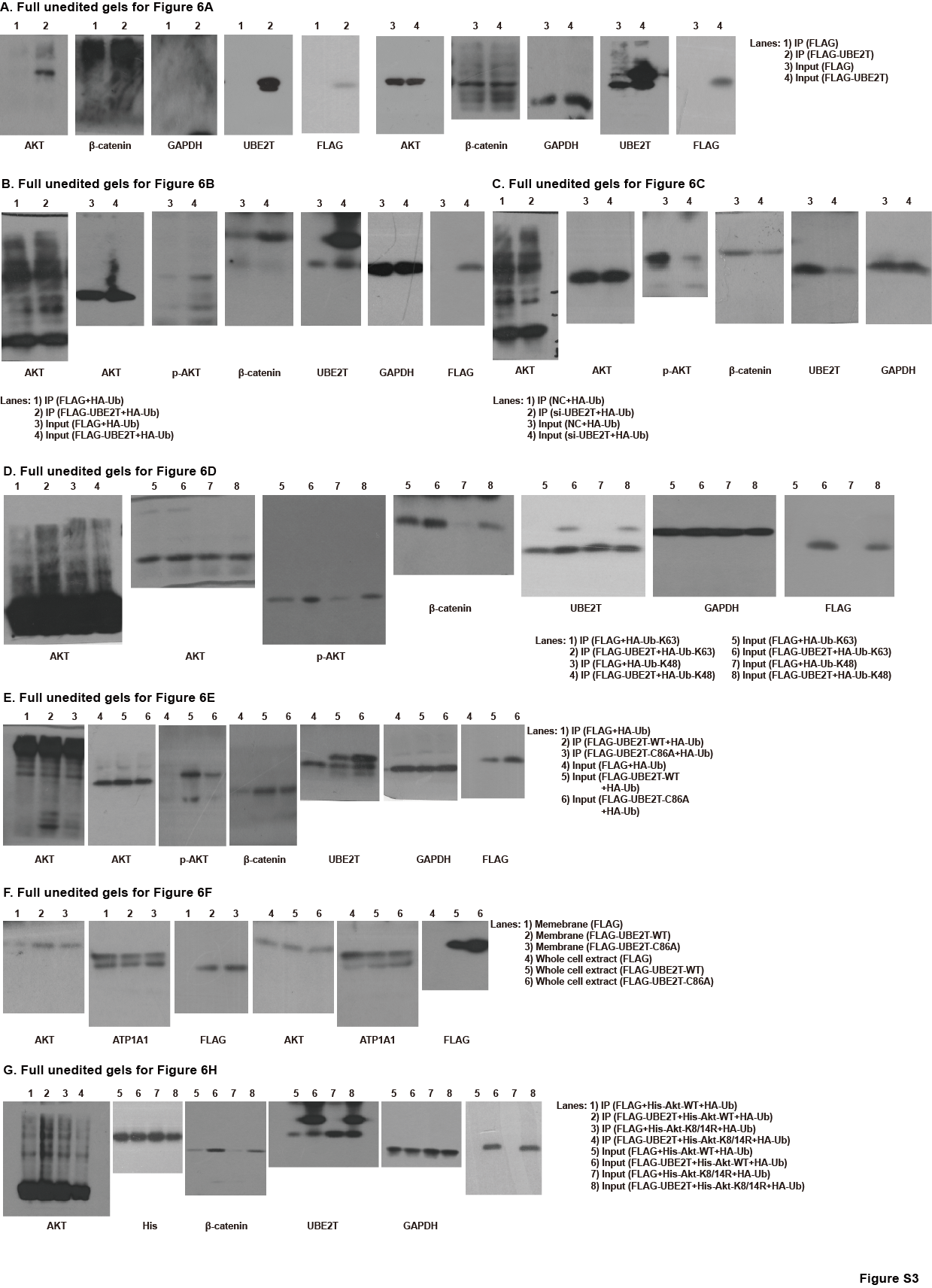
**

**
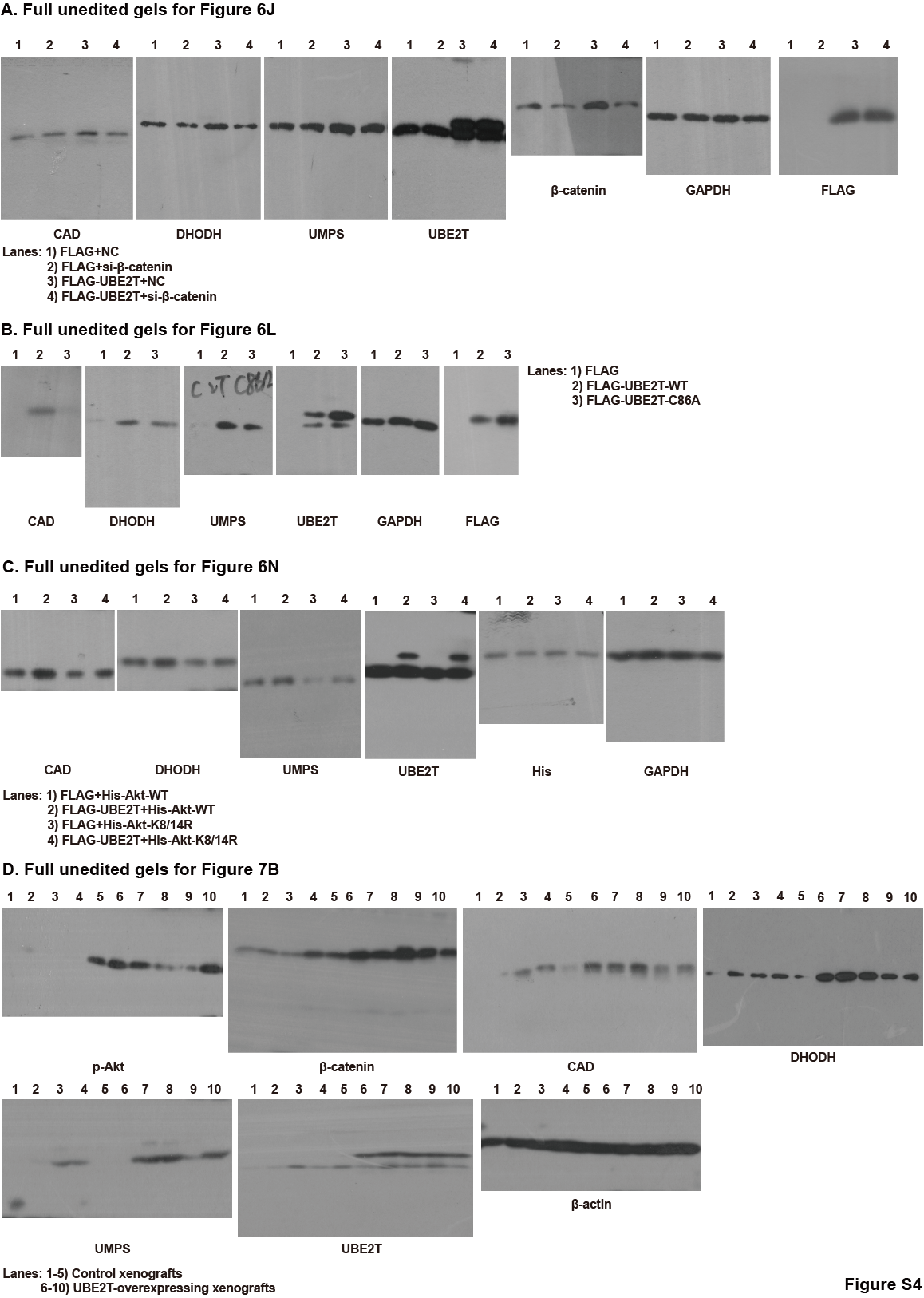
**

**
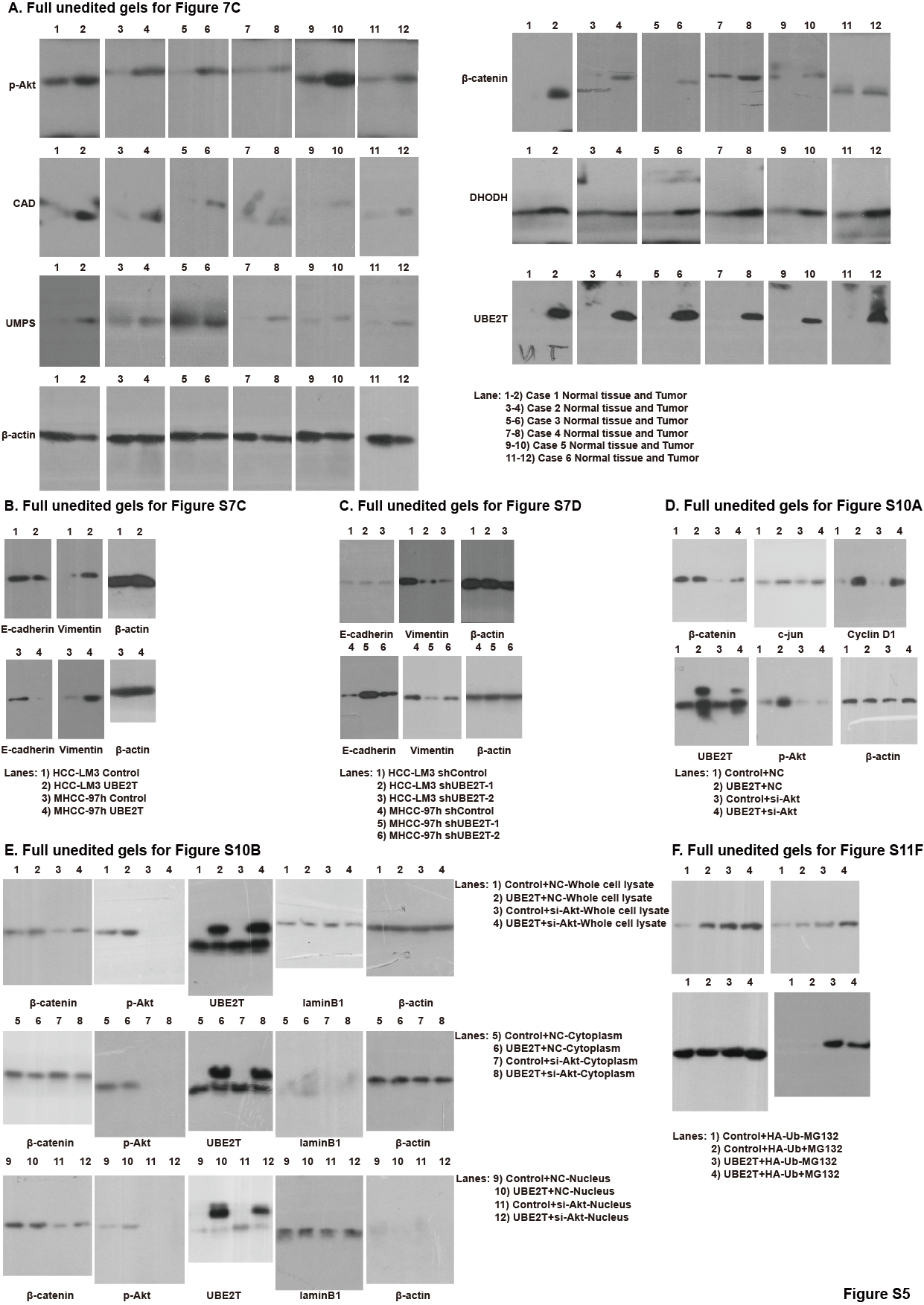
**

**Supplementary Figures**

**
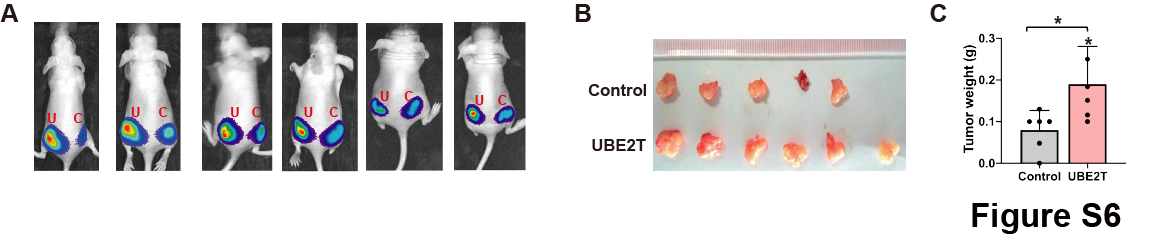
**

**Fig. S6 UBE2T promotes HCC proliferation in *vivo*. a** Bioluminescence images of the mice injected subcutaneously with UBE2T-overexpressing and control MHCC-97H cells. The luminescence signal is represented by an overlaid false-color image with the signal intensity indicated by the scale. **b** The xenografts from panel **(a)** were shown. **c** Tumor weights of the removed xenografts from panel **(a)**.

**
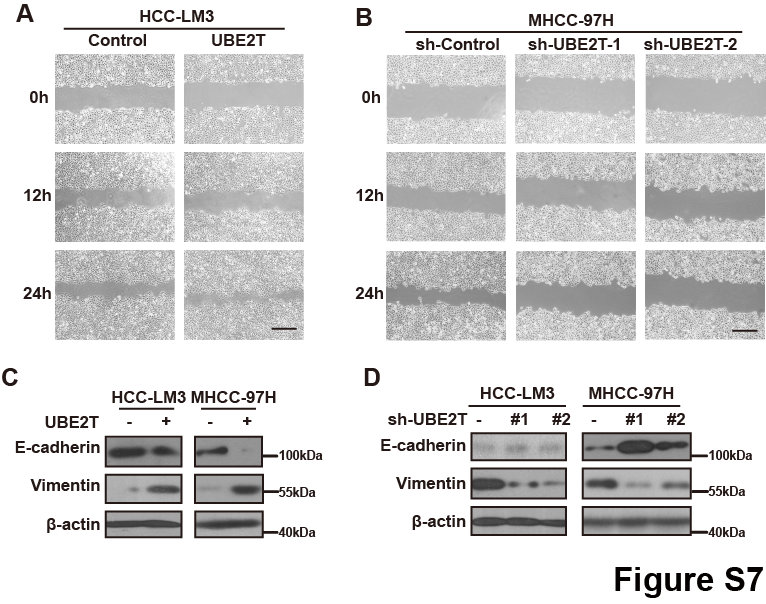
**

**Fig. S7 UBE2T promotes migration and EMT in HCC cells. a-b** Wound healing assays to assess the migration of UBE2T-overexpressing and control HCC-LM3 cells (**a)**, UBE2T-silencing and control MHCC-97H cells **(b)**. **c-d** WB was used to assess EMT markers in UBE2T-overexpressing and control, or UBE2T-silencing and control HCC-LM3/MHCC-97H cells.

**
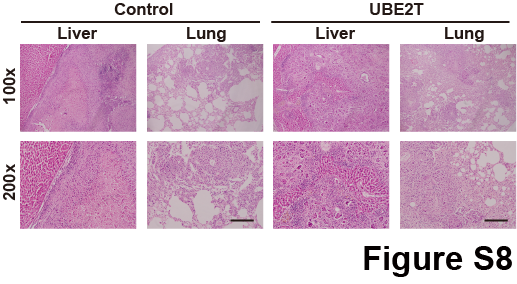
**

**Figure S8. The metastases in livers and lungs of the mice from Fig. 2e were examined by H&E staining.**

**
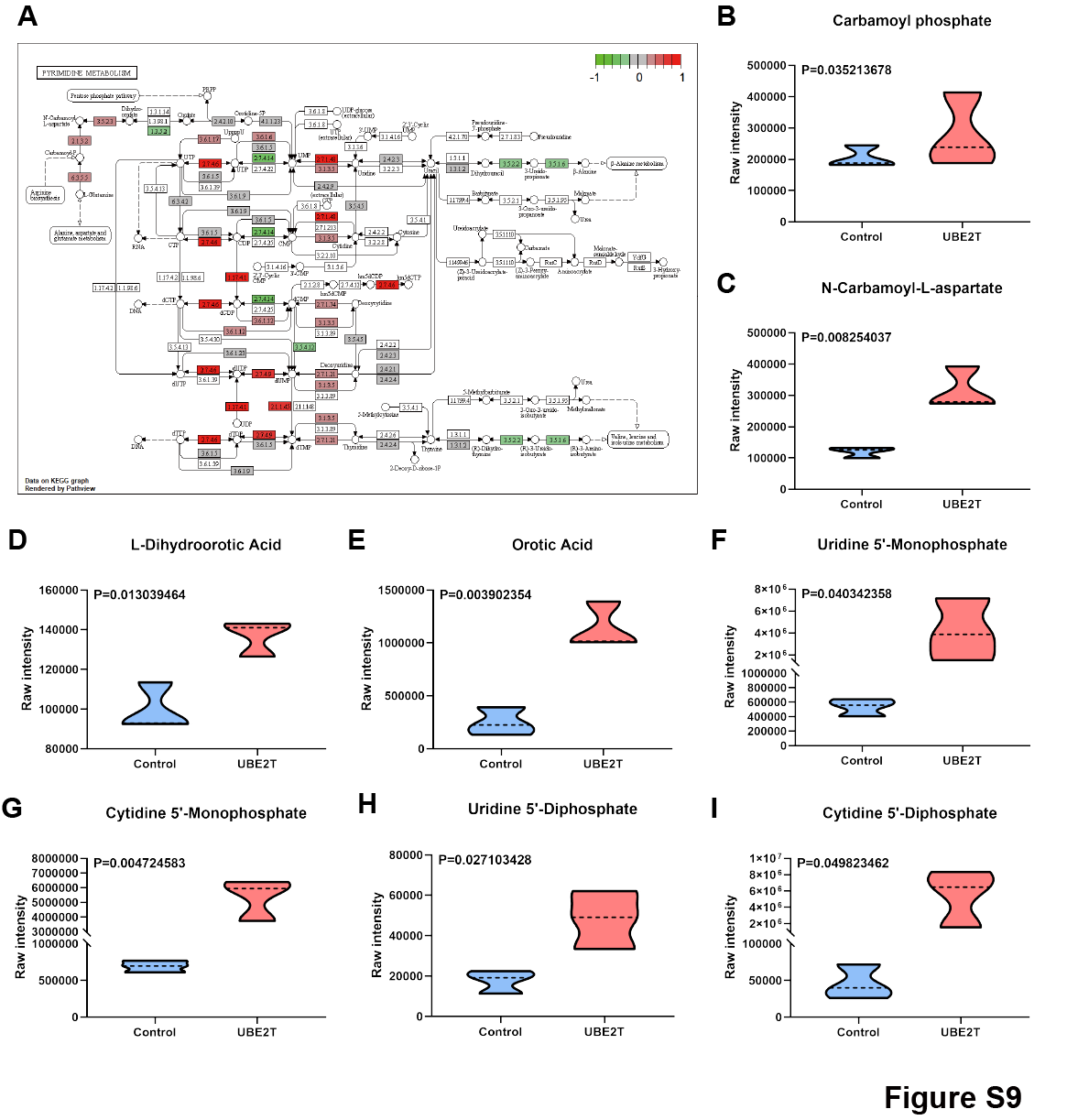
**

**Figure S9. KEGG pathview and LC/MS-MS metabolomics profiling indicated that UBE2T promoted pyrimidine metabolism. a** Pyrimidine metabolism pathways from the KEGG database. Genes with colors are considered to be differently expressed between UBE2T-high and -low phenotype. Red: genes upregulated in UBE2T-high phenotype. Green: genes downregulated in UBE2T-high phenotype. **b-i** Raw intensity of products from pyrimidine metabolism in HCC-LM3 cells stably transducted with control and UBE2T-overexpressed lentivirus.


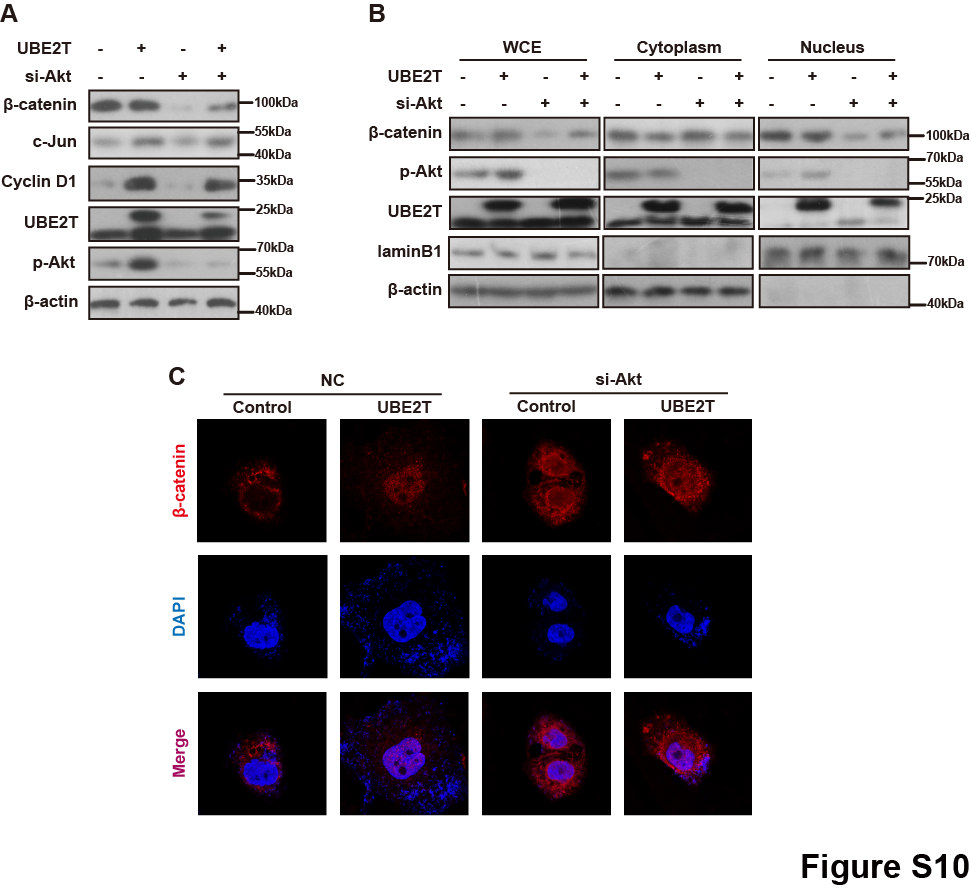


**Figure S10. Knockdown of Akt impairs the effect of UBE2T on activation of β-catenin. a** UBE2T-overexpressing and control HCC-LM3 were transfected with si-Akt. Total cell lysates were detected for the indicated proteins. **b** Cell were treated as **(a)** and then collected for subcellular protein extraction, following by WB to detect β-catenin and p-Akt. **c** Cells were treated as panel **(a)**. Representative images of immunofluorescence staining for β-catenin (Red) are shown. Scale bar=200μm.

**
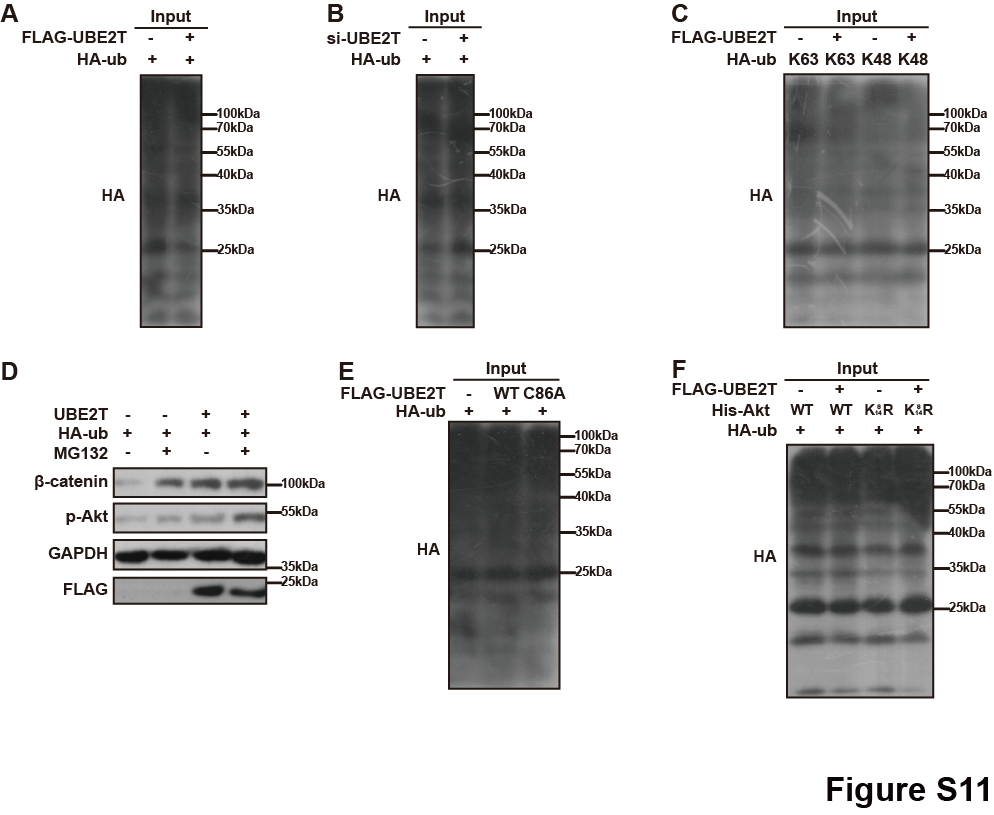
**

**Figure S11. UBE2T activates Akt/β-catenin pathway via regulating Akt K63-ubiquitination. a** Transfection efficiency of HA-ub detected by WB in Figure 6b. **b** Transfection efficiency of HA-ub detected by WB in Figure 6c. **c** Transfection efficiency of HA-ub detected by WB in Figure 6d. **d** Transfection efficiency of HA-ub detected by WB in Figure 6e. **e** Transfection efficiency of HA-ub detected by WB in Figure 6h. **f** UBE2T-overexpressing and control HCC-LM3 cells were transfected with HA-ub vector, and then treated with or without MG132, following by WB to detect β-catenin and p-Akt.

**Table S1. Antibodies used in this study.**

| Product | Vendor | Cat No. | Application |
| --- | --- | --- | --- |
| UBE2T | Proteintech | 10105-2-AP | WB, IHC |
| β-actin | Rui Antibody Biotechnology | RM2001 | WB |
| PARP | Cell Signaling Technology | 9532 | WB |
| Cleaved PARP | Cell Signaling Technology | 9548 | WB |
| Caspase-3 | Cell Signaling Technology | 9662 | WB |
| Cleaved Caspase-3 | Cell Signaling Technology | 9664 | WB |
| Vimentin | Cell Signaling Technology | 5741 | WB |
| E-cadherin | Cell Signaling Technology | 3195 | WB |
| CAD | Proteintech | 16617-1-AP | WB, IHC |
| DHODH | Proteintech | 14877-1-AP | WB, IHC |
| UMPS | Proteintech | 14830-1-AP | WB, IHC |
| GAPDH | Rui Antibody Biotechnology | RM2002 | WB |
| Akt | Cell Signaling Technology | 4691 | WB |
| p-Akt | Cell Signaling Technology | 4060 | WB, IHC |
| GSK3β | Cell Signaling Technology | 12456 | WB |
| p-GSK3β | Cell Signaling Technology | 5558 | WB |
| STAT3 | Cell Signaling Technology | 9139 | WB |
| p-STAT3 | Cell Signaling Technology | 9145 | WB |
| IKKβ | Cell Signaling Technology | 8943 | WB |
| p-IKKβ | Cell Signaling Technology | 2694 | WB |
| mTOR | Cell Signaling Technology | 2983 | WB |
| p-mTOR | Cell Signaling Technology | 5536 | WB |
| p38 | Cell Signaling Technology | 8690 | WB |
| p-p38 | Cell Signaling Technology | 4511 | WB |
| Smad2/3 | Cell Signaling Technology | 8685 | WB |
| p-Smad2 | Cell Signaling Technology | 18338 | WB |
| c-Jun | Cell Signaling Technology | 9165 | WB |
| Cyclin D1 | Cell Signaling Technology | 55506 | WB |
| β-catenin | Cell Signaling Technology | 8480 | WB, IHC, IF |
| ATP1A1 | Proteintech | 14418-1-AP | WB |
| His | Rui Antibody Biotechnology | RM1001 | WB |
| myc | Rui Antibody Biotechnology | RM1003 | WB |
| HA | Rui Antibody Biotechnology | RM1004 | WB, IP |

**Table S2. Primers used in this study.**

| Genes | Primer sequence |
| --- | --- |
| CAD | F AGTGGTGTTTCAAACCGGCAT |
|  | R CAGAGGATAGGTGAGCACTAAGA |
| DHODH | F GTTCTGGGCCATAAATTCCGA |
|  | R TCTGGGTCTAGGGTTTCCTTC |
| UMPS | F GTGTGTGGAGTGCCTTATACAG |
|  | R CCTTCTACAAGACGCTTAGTTCC |
| β-catenin | F AAAGCGGCTGTTAGTCACTGG |
|  | R CGAGTCATTGCATACTGTCCAT |
| β-actin | F CATGTACGTTGCTATCCAGGC |
|  | R CTCCTTAATGTCACGCACGAT |

**Table S3. Clinicopathological characteristics of 38 HCC patients in Figure 7.**

| Feature N | | |  |  |
| --- | --- | --- | --- | --- |
| Gender  Male  Female | 30  8 | | |  |
| Age  ≤55  >55 | 26  12 | | |  |
| Edmondson Grade  Ⅰ+Ⅱ  Ⅲ+Ⅳ | 29  9 | | |  |
| Liver cirrhosis  With  without | 22  16 | | |  |
| HBV  Positive  negative | 32  6 | | |  |
| Envelop |  | | |  |
| With  Without | 25  13 | | |  |
| ALB(g/L）  ≤40  >40 | 23  15 | | |  |
| AST(U/L)  ≤40:  >40 | 16  22 | | |  |
| ALT(U/L)  ≤45  >45 | 18  20 | | |  |
| AFP (μg/L)  ≤20  >20 | 9  29 | | |  |
| Serum total bilirubin(μmol/L)  ≤17  >17 | | 17  21 | | |
| Tumor Size(cm) |  | | |  |
| ≤5  >5 | 7  31 | | |  |
| Relapse |  | | |  |
| Yes  No | 24  14 | | |  |
| Portal vein tumor thrombus  No  Yes | 21  17 | | |  |
| No. tumor  Solitary  Multiple | 32  6 | | |  |
| Metastasis |  | | |  |
| Yes  No | 5  33 | | |  |
| BCLC stage  A  B+C+D | 28  10 | | |  |
